# Use of Digital Health Technology Among Older Adults With Cancer in the United States: Findings From a National Longitudinal Cohort Study (2015-2021)

**DOI:** 10.2196/46721

**Published:** 2023-05-31

**Authors:** Weijiao Zhou, Youmin Cho, Shaomei Shang, Yun Jiang

**Affiliations:** 1 School of Nursing Peking University Beijing China; 2 School of Biomedical Informatics The University of Texas Health Science Center at Houston Houston, TX United States; 3 School of Nursing University of Michigan Ann Arbor, MI United States

**Keywords:** digital health, technology, older adults, cancer, survivorship, cancer survivor, older cancer survivors, digital health technology

## Abstract

**Background:**

Despite the benefits of digital health technology use, older adults with cancer (ie, aged 65 years) have reported challenges to technology adoption. However, there has been a lack of a good understanding of their digital health technology use patterns and the associated influential factors in the past few years.

**Objective:**

This study aimed to examine the trends in and factors associated with digital health technology use among older adults with cancer.

**Methods:**

The National Health and Aging Trends Study (NHATS) data set is a national longitudinal cohort study with annual survey waves of Medicare beneficiaries 65 years and older. Participants were community-dwelling older adults who self-reported previous or current cancer diagnoses in each round. The study sample size of each round ranged from 1996 (2015) to 1131 (2021). Digital health technology use was defined as using the internet or online in the last month to order or refill prescriptions, contact medical providers, handle Medicare or other insurance matters, or get information about their health conditions. The association of sociodemographics, clinical factors (self-rated health, chronic conditions, difficulties in activities of daily living, dementia, anxiety, and depression), and physical function (Short Physical Performance Battery and grip strength) with digital health technology use was examined using design-based logistic regression. All statistical analyses accounted for the complex sample design.

**Results:**

The prevalence of any digital health technology use increased from 36% in 2015 to 45% in 2019. In 2020-2021, which was amid the COVID-19 pandemic, it ranged from 51% to 52%. In terms of each digital health technology use behavior, in 2015, overall, 28% of older cancer survivors used digital health technology to obtain health information, followed by contacting clinicians (19%), filling prescriptions (14%), and handling insurance (11%). Greater use of digital health technology was associated with younger age, being White, having a college or higher education, having a higher income, having more comorbidities, nondementia, and having a higher gait speed.

**Conclusions:**

Digital health technology use in older adults with cancer has gradually increased, particularly during the COVID-19 pandemic. However, socioeconomic and racial disparities have remained in older cancer survivors. Additionally, older adults with cancer may have some unique features associated with digital health technology use; for example, their use of digital health may be increased by their comorbidities (ie, health care needs) and reduced by their frailty.

## Introduction

In 2022, there were over 18 million cancer survivors in the United States, which account for approximately 5% of the entire population [1]. Among those cancer survivors, 67% are currently aged 65 years or older, and it is estimated to increase to 74% by 2040 [1]. During and after cancer treatment, cancer survivors struggle with multiple acute or chronic symptoms related to their treatments or the disease [2]. In recent years, digital health technologies, such as electronic communications with clinicians or telehealth visits, have been increasingly used to facilitate health care delivery. It has been demonstrated that digital health technology use among patients with and survivors of cancer, such as electronic communication with health care providers, reduces their symptom distress and emergency department admissions and improves survival rates [3-6]. Furthermore, patients with and survivors of cancer have experienced improved well-being and better patient satisfaction while using digital health technology [7-10].

A body of literature has pointed out that older adults (aged 65 years or older) have shown a significantly lower use of digital health technology than individuals in other age groups [11-13], which may be because older adults tend to prefer direct in-person relationships with their health care providers, having potential concerns about eroding patient-provider trust and information privacy and security while using digital health technology [12,13]. According to Levine et al’s [14,15] research from 2016 and 2018, the everyday use of technology by older adults, including web-based shopping or banking, was lower than that of the general population. Furthermore, their use of digital health technology was even lower than their use of everyday technology, and this decreased gradually as their health declined [14,15]. Regarding older adults with cancer, previously published literature supports low use of everyday technology [16], but it also reveals a slight increase in use since the COVID-19 pandemic [17]. A secondary analysis of nationally representative survey data in the United States revealed that cancer survivors aged 65 years or older were significantly less likely to use digital health technology to communicate with their providers than younger survivors [18]. Considering the increasing number of older adults with cancer in the United States and their high level of cancer care needs, they can be the potential major users to be benefited from the use of digital health technology [1,13,18]. A few qualitative studies revealed that older adults with cancer possess a positive attitude toward using digital health technology, particularly after experiencing telehealth during the COVID-19 pandemic [19-21]. However, there is a lack of a good understanding of the pattern of digital health technology use among older adults with cancer, as measured quantitatively and longitudinally in the past few years, considering the impact of the COVID-19 pandemic.

Therefore, the aims of this study were to (1) examine the trends in digital health technology use over time and compare the patterns with everyday technology use (from 2015 to 2021) and (2) identify factors associated with digital health technology use among older adults with cancer. Through analyzing the data from the National Health and Aging Trends Study (NHATS), this study was expected to obtain general insights to inform researchers and health care providers to enhance sustainable oncology care delivery to older adults with cancer.

## Methods

### Data Sources and Study Population

The NHATS is a longitudinal cohort study with annual survey waves of Medicare beneficiaries aged 65 years and older living in the community, residential care, and nursing homes [22]. Data had been collected since 2011 (round 1) and replenished in 2015 (round 5). In both rounds 1 and 5, participants were selected through a stratified 3-stage sample design, with oversampling of older persons and Black non-Hispanic individuals [23]. Each round produced an analytical weight that accounted for differential probabilities of selection and nonresponse. In rounds 1-9, the interview was conducted in person, whereas in round 10, the NHATS interview was conducted by telephone because of the COVID-19 pandemic.

**Figure 1:**
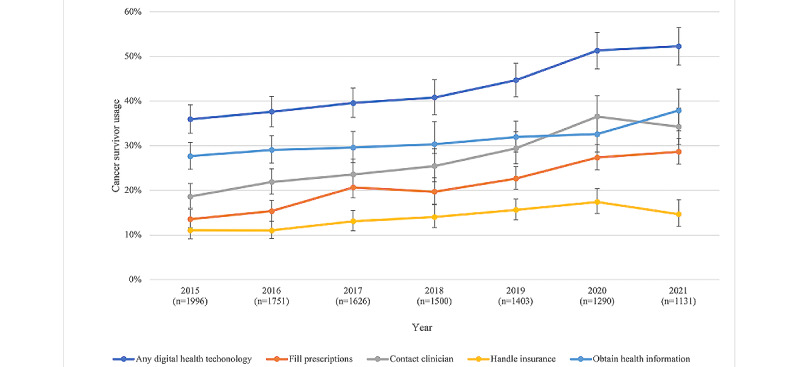
Change in digital health technology use (weighted). Error bars indicate 95% CIs of the weighted percentages. Individuals who had cancer in previous rounds and were newly diagnosed with cancer in each round were included in the analyses.

We used cohort data from round 5 to round 11. In round 5, a total of 8334 older adults completed the interviews, and these respondents have been annually reinterviewed until 2021 (round 11). The weighted response rate (rounds 5-11) ranged from 73.6% to 96.0%. For each round, we included participants who were community-dwelling and with self-reported cancer diagnosis in that round or prior rounds. The study sample size of each round is shown in Figure 1, ranging from 1996 (round 5) to 1131 (round 11). For the second aim, participants with missing data of sociodemographic, clinical, and physical factors were excluded. A complete case analysis was conducted.

### Ethics Approval

The NHATS was approved by the Johns Hopkins Bloomberg School of Public Health institutional review board. All participants provided informed consent that described the purpose of a nationally representative survey study designed to benefit numerous researchers. This study was exempt from an institutional ethical review because it involved a publicly available, deidentified data set.

### Measures

Digital health technology use was measured by 4 items [14,15]. Participants were asked whether they used the internet or went online in the last month to (1) order or refill prescriptions, (2) contact medical providers, (3) handle Medicare or other insurance matters, or (4) get information about their health conditions. Any digital health technology use was coded as “Yes” when any of the above 4 digital health use behaviors was confirmed. Everyday technology use was coded as “Yes” if participants reported using internet or going online for any reason besides email or texting in the last month. In terms of each type of everyday technology use, participants were asked whether they used the internet or went online in the last month to (1) shop for groceries or personal items (yes or no), (2) pay bills or do banking (yes or no), or (3) visit social network sites (yes or no) [14,15].

Sociodemographic characteristics included (1) age (65-69, 70-74, 75-79, 80-84, 85-89, ≥90 years), (2) sex (female or male), (3) race and ethnicity (non-Hispanic White, non-Hispanic Black, Hispanic, or other), (4) marital status (married or partnered, or not), (5) educational level (less than high school, high school graduate, some college, college graduate, or higher), and (6) annual income (<US $15,000; $15,000-$29,999; $30,000-$44,999; $45,000-$60,000; or >$60,000). Clinical factors, mainly related to self-reported clinical diagnoses and health status, included the following: (1) self-rated health, assessed as excellent, very good, good, fair, or poor; (2) number of chronic conditions, assessed by self-reports of the following chronic conditions: heart attack, heart disease, hypertension, arthritis, osteoporosis, diabetes, lung diseases, and stroke, which ranged from 0 to 8; (3) number of difficulties in activities of daily living (ADL), assessed by validated items assessing whether they had difficulty with the following activities: eating, showering, or bathing, using the toilet, getting dressed, getting out of bed, and getting around inside the home, which ranged from 0 to 6 [24]; (4) diagnosis of dementia, which was self-reported as yes or no; (5) anxiety and depression, assessed by the sum score of the 2 items of the Patient Health Questionnaire-2 and 2 items of the Generalized Anxiety Disorder-2 scale [25,26], which generates a total depression and anxiety score ranging from 4 to 16, with a higher score indicating poorer mental health. Physical functions, mainly related to physical abilities measured by objective methods, included (6) physical performance, assessed by Short Physical Performance Battery (SPPB), which included tests of gait speed, chair stands, and balance activities [27] and rated each from 0 to 4 and generated the total SPPB score ranging from 0 to 12, with a higher score indicating better physical performance; and (7) grip strength, measured twice by a dynamometer (using the highest value), scored using quartiles of the NHATS sample distribution [28], which ranged from 0 to 4, with a higher score indicating better grip strength.

### Statistical Analysis

Stata SE (version 17.0; StataCorp) was used for statistical analyses. For aim 1, the complex sample design (ie, stratification and primary sampling units) and sampling weights in each round (rounds 5-11) were accounted for in the analyses. The weighed percentage and 95% CIs were calculated to summarize the digital health technology use and everyday technology use in each round. For aim 2, the complex sample design was also accounted for in the analyses. Descriptive statistics (ie, percentages for categorical variables and means and SEs for continuous variables) were calculated to summarize the digital health technology use and sociodemographic, clinical, and physical function factors. Bivariate analyses were used to compare the factors of different groups (ie, none vs any digital health technology use); bivariate differences were assessed with Rao-Scott chi-square tests for categorical variables and design-based *F* tests for continuous variables. Design-based logistic regression analyses were performed to examine the association between any digital health technology use (yes or no, dependent variable) and (1) sociodemographic factors (model 1); (2) sociodemographic and clinical factors (model 2); and (3) sociodemographic, clinical, and physical function factors (model 3). We conducted analyses using data from round 5 (n=1760) and the recent round 9 (n=1246) and round 11 (n=965). Round 10 was not analyzed because of missing data of physical function–related variables. The adjusted odds ratios (ORs) and 95% CIs were calculated.

## Results

### Characteristics of Study Population

Table 1 shows the characteristics of the study population collected in 2015. More than 50% of American older adults with cancer were 75 years and older. Most were female (50.2%); non-Hispanic White (88.2%); married or partnered (61.1%); reported excellent, very good, or good health (75.5%); did not obtain college degree (67.4%); and reported comorbidity (81.7%) and no ADL limitation (66.4%). Their average anxiety and depression score was 5.7 (range 4-16), SPPB score was 7.0 (range 0-12), and grip strength score was 2.2 (range 0-4). Individuals who reported any use of digital health technology were more likely to be younger, White, married or partnered, have higher education and income, better self-related health status, less comorbidities, less ADL limitations, lower anxiety and depression score, higher physical performance, and higher grip strength score (*P*<.001).

**Table 1:** Characteristics of older adults with cancer by digital health technology use in the United States, 2015 (N=1760).^a^

Characteristic			Overall		Digital health technology use				*P* value^b^	
					None (n=1239)		Any (n=521)			
**Age (years), %^c^ (95% CI)**										<.001
	65-69	20.9 (17.9-24.2)		15.9 (12.5-20.0)		29.4 (24.6-34.8)				
	70-74	26.9 (24.8-29.2)		22.7 (20.0-25.6)		34.1 (29.9-38.6)				
	75-79	21.6 (19.4-24.0)		22.1 (19.2-25.2)		20.9 (17.5-24.8)				
	80-84	16.3 (14.5-18.2)		20.1 (17.7-22.7)		9.8 (7.9-12.3)				
	85-89	9.7 (9.6-11.0)		12.7 (11.1-14.3)		4.7 (3.5-6.4)				
	≥90	4.5 (3.7-5.4)		6.6 (5.4-8.0)		1.0 (0.5-1.9)				
Female, % (95% CI)			50.2 (47.6-52.9)		52.7 (49.8-55.5)		46.1 (40.6-52.9)		.05	
**Race and ethnicity, % (95% CI)**										<.001
	Non-Hispanic White	88.2 (85.9-90.1)		84.4 (81.3-87.1)		94.5 (92.2-96.2)				
	Non-Hispanic Black	5.0 (4.3-5.9)		6.5 (5.4-7.9)		2.5 (1.8-3.6)				
	Hispanic	3.7 (2.6-5.2)		5.3 (3.7-7.5)		0.9 (0.3-2.6)				
	Other	3.1 (2.0-4.7)		3.8 (2.5-5.6)		2.0 (0.9-4.4)				
Married or partnered, % (95% CI)			61.1 (58.2-63.9)		54.3 (51.0-57.6)		72.5 (68.2-76.5)		<.001	
**Education level, % (95% CI)**										<.001
	Less than high school	14.5 (12.4-16.8)		21.4 (18.7-24.4)		2.7 (1.3-5.4)				
	High school graduate	26.2 (23.6-29.1)		33.0 (30.0-36.2)		14.7 (11.3-18.9)				
	Some college	26.7 (23.9-29.7)		25.4 (22.1-28.9)		29.0 (24.8-33.5)				
	College graduate or higher	32.6 (28.6-36.9)		20.2 (17.2-23.6)		53.6 (47.1-60.0)				
**Annual income (US $), % (95% CI)**										<.001
	<15,000	10.5 (9.0-12.3)		15.1 (13.0-17.4)		2.8 (1.6-4.9)				
	15,000-29,999	20.5 (18.3-23.0)		26.8 (23.8-30.0)		9.9 (7.5-13.0)				
	30,000-44,999	18.2 (16.3-20.2)		19.8 (17.5-22.3)		15.5 (11.9-20.0)				
	45,000-60,000	12.1 (10.3-14.0)		11.5 (9.2-14.3)		13.0 (9.7-17.1)				
	>60,000	38.7 (34.6-42.9)		26.8 (22.8-31.1)		58.9 (52.6-64.8)				
**Self-rated health, % (95% CI)**										<.001
	Excellent	12.3 (10.3-14.6)		9.4 (7.8-11.4)		17.2 (13.0-22.3)				
	Very good	28.8 (26.2-31.5)		24.3 (21.5-27.3)		36.3 (31.8-41.1)				
	Good	34.4 (31.9-37.1)		36.5 (33.2-40.0)		30.9 (26.6-35.5)				
	Fair	18.0 (15.7-20.5)		21.4 (18.9-24.2)		12.1 (8.9-16.2)				
	Poor	6.5 (5.0-8.4)		8.3 (6.4-10.7)		3.5 (2.0-6.3)				
**Number of comorbidities, % (95% CI)**										.02
	0	8.3 (6.8-10.0)		7.4 (5.7-9.5)		9.8 (7.1-13.4)				
	1-2	49.4 (46.4-52.4)		47.1 (43.8-50.4)		53.3 (47.7-58.9)				
	3-8	42.3 (39.4-45.3)		45.5 (42.5-48.6)		36.8 (32.1-41.9)				
**Number of ADL^d^ limitations, % (95% CI)**										<.001
	0	66.4 (63.0-69.7)		61.7 (58.3-64.9)		74.5 (69.6-78.8)				
	1-2	22.6 (20.3-25.2)		24.2 (21.2-27.4)		20.0 (16.5-24.1)				
	3-6	10.0 (9.1-13.1)		14.2 (11.8-16.9)		5.5 (3.7-7.9)				
Dementia, % (95% CI)			3.7 (2.8-4.8)		5.6 (4.3-7.2)		0.5 (0.2-0.9)		<.001	
Anxiety and depression score, mean (95% CI)			5.7 (5.6-5.9)		6.0 (5.8-6.2)		5.3 (5.1-5.5)		<.001	
**SPPB,^e^ mean (95% CI)**			7.0 (6.8-7.3)		6.2 (6.0-6.5)		8.4 (8.1-8.7)		<.001	
	Gait speed	2.4 (2.3-2.5)		2.1 (2.0-2.2)		2.9 (2.8-3.0)		<.001		
	Balance test	2.4 (2.4-2.5)		2.2 (2.1-2.3)		2.9 (2.8-3.0)		<.001		
	Chair test	2.2 (2.1-2.3)		1.9 (1.8-2.0)		2.6 (2.5-2.7)		<.001		
Grip strength score, mean (95% CI)			2.2 (2.1-2.3)		2.1 (2.0-2.2)		2.5 (2.4-2.7)		<.001	

^a^National estimates based on the complex survey design.

^b^*P* values compare individuals reported any versus no use of digital health.

^c^Percentages represent the weighted prevalence, and they may not sum to 100 due to rounding.

^d^ADL: activities of daily living.

^e^SPPB: Short Physical Performance Battery.

### Prevalence of Digital Health and Everyday Technology Use: 2015-2021

The prevalence of any digital health technology use among older adults with cancer increased from 36% in 2015 to 45% in 2019 and continued increasing in 2020 and 2021, in the amid the COVID-19 pandemic, to 51% and 52%, respectively (see Figure 1). In terms of each digital health technology use behavior, the overall use tended to increase over time, from 2015 to 2021 (see Figure 1). However, the use of technology for contacting clinicians or filling prescriptions had a more dramatic increase than other 2 types of use, obtaining health information and handling insurance. The use for obtaining health information had the highest prevalence in almost all years, whereas the use for contacting clinician had the most dramatic increase (from 19% to 34%). In 2020, with the start of the COVID-19 pandemic, the prevalence of use for contacting clinicians (37%) increased very fast, becoming even higher than the use for obtaining health information (33%). Interestingly, in the following year (2021), the use for contacting clinicians dropped slightly to 34%, whereas the use for obtaining health information increased to 38%, becoming the top one use again.

**Figure 2:**
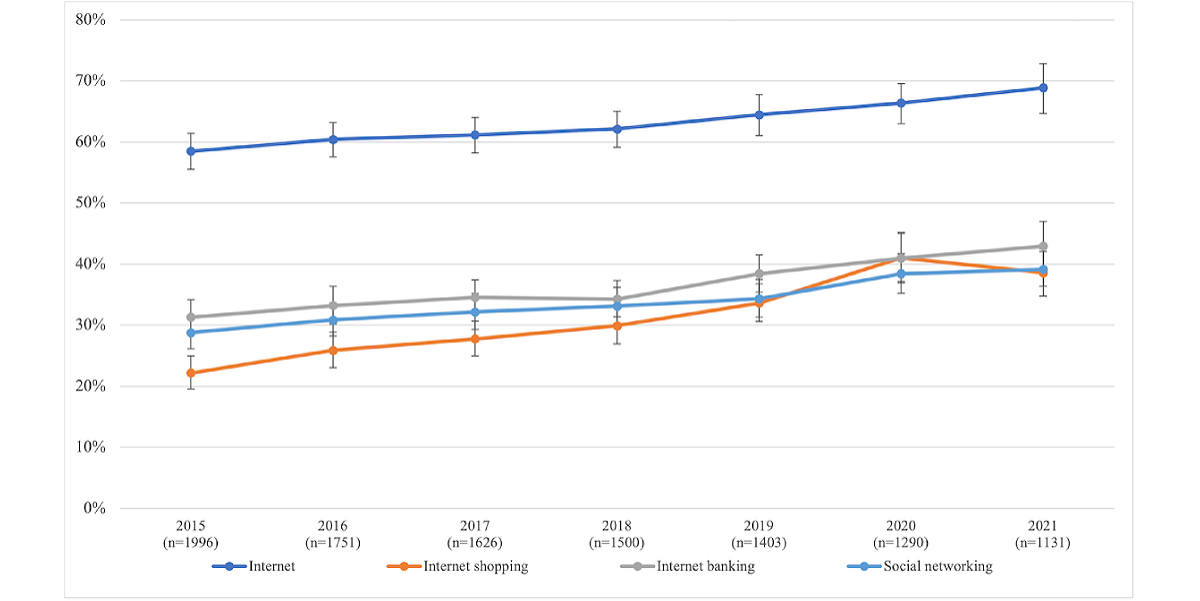
Change in everyday health technology use (weighted). Error bars indicate 95% CIs of the weighted percentages. Individuals who had cancer in previous rounds and were newly diagnosed with cancer in each round were included in the analyses.

In general, the proportion of older adults with cancer who used the internet increased gradually from 59% in 2015 to 69% in 2021 (see Figure 2). In terms of each everyday technology use behavior, the overall use tended to increase over time, from 2015 to 2021 (see Figure 2).

### Factors Associated With Any Digital Health Technology Use

Table 2 shows the results of design-based logistic regressions. Model 1 showed that variables associated with greater use of digital health were younger age, being White, having a college or higher education, and having higher income. In model 2, when adding clinical factors to the logistic regression model, the above factors remained as being statistically significant. Additionally, more comorbidities (adjusted OR 1.14, 95% CI 1.02-1.27), fewer ADL limitations (adjusted OR 0.88, 95% CI 0.78-0.99), and nondementia (adjusted OR 0.15, 95% CI 0.07-0.34) were also associated with higher use of digital health. When physical function factors were added to model 3, ADL limitation was no longer significantly associated with digital health use, whereas higher gait speed was associated with greater digital health use (adjusted OR 1.20, 95% CI 1.02-1.40) (see Multimedia Appendix 1 for results from rounds 9 and 11).

**Table 2:** Weighted estimates of odds ratio in logistic regression models, 2015.^a^

Characteristic			Any use of digital health, aOR^b^ (95% CI)				
			Model 1		Model 2		Model 3
**Age (years)**							
	65-69	Reference		Reference		Reference	
	70-74	0.75 (0.48-1.18)		0.73 (0.45-1.18)		0.77 (0.46-1.26)	
	75-79	0.64 (0.40-1.03)		0.66 (0.38-1.12)		0.72 (0.41-1.28)	
	80-84	*0.32* (*0.20-0.52*)^c^		*0.32* (*0.19-0.53*)		*0.37* (*0.21-0.66*)	
	85-89	*0.26* (*0.15-0.45*)		*0.28* (*0.16-0.49*)		*0.33* (*0.17-0.64*)	
	≥90	*0.12* (*0.05-0.28*)		*0.13* (*0.05-0.32*)		*0.18* (*0.06-0.48*)	
Female			1.08 (0.78-1.49)		1.06 (0.75-1.49)		1.04 (0.69-1.56)
**Race and ethnicity**							
	White	Reference		Reference		Reference	
	Black	*0.53* (*0.34-0.84*)		*0.54* (*0.34-0.87*)		*0.57* (*0.35-0.93*)	
	Hispanic	*0.27* (*0.10-0.75*)		*0.27* (*0.10-0.71*)		*0.29* (*0.12-0.70*)	
	Other	0.58 (0.21-1.66)		0.62 (0.23-1.72)		0.67 (0.25-1.81)	
Married or partnered			1.06 (0.76-1.47)		1.08 (0.76-1.52)		1.05 (0.74-1.49)
**Education level**							
	Less than high school	*0.10* (*0.05-0.22*)		*0.10* (*0.05-0.22*)		*0.11* (*0.05-0.24*)	
	High school graduate	*0.25* (*0.18-0.34*)		*0.24* (*0.17-0.34*)		*0.25* (*0.18-0.36*)	
	Some college	*0.53* (*0.39-0.72*)		*0.52* (*0.38-0.71*)		*0.55* (*0.40-0.75*)	
	College graduate or higher	Reference		Reference		Reference	
**Annual income (US $)**							
	<15,000	Reference		Reference		Reference	
	15,000-29,999	1.40 (0.77-2.53)		1.37 (0.75-2.49)		1.42 (0.79-2.55)	
	30,000-44,999	*2.32* (*1.13-4.74*)		*2.25* (*1.06-4.75*)		*2.27* (*1.09-4.75*)	
	45,000-60,000	*2.52* (*1.14-5.56*)		*2.53* (*1.13-5.68*)		*2.52* (*1.15-5.54*)	
	>60,000	*3.13* (*1.57-6.24*)		*2.81* (*1.35-5.87*)		*2.80* (*1.35-5.80*)	
**Self-rated health**							
	Excellent	N/A^d^		Reference		Reference	
	Very good	N/A		0.83 (0.53-1.31)		0.84 (0.54-1.33)	
	Good	N/A		0.76 (0.45-1.28)		0.81 (0.48-1.37)	
	Fair	N/A		0.70 (0.35-1.37)		0.74 (0.38-1.47)	
	Poor	N/A		0.88 (0.31-2.49)		0.99 (0.34-2.91)	
Number of comorbidities			N/A		*1.14* (*1.02-1.27*)		*1.15* (*1.04-1.27*)
Number of ADL^e^ limitations			N/A		*0.88* (*0.78-0.99*)		0.93 (0.81-1.06)
Dementia			N/A		*0.15* (*0.07-0.34*)		*0.16* (*0.07-1.06*)
Anxiety and depression			N/A		0.96 (0.88-1.05)		0.96 (0.88-1.05)
Gait speed			N/A		N/A		*1.20* (*1.02-1.40*)
Balance test			N/A		N/A		1.10 (0.94-1.29)
Chair test			N/A		N/A		0.94 (0.83-1.08)
Grip strength score			N/A		N/A		0.97 (0.84-1.12)

^a^Models were adjusted for the complex survey design.

^b^aOR: adjusted odds ratio.

^c^Italicized values mean *P*<.05.

^d^N/A: not applicable.

^e^ADL: activities of daily living.

## Discussion

### Principal Findings

This study, to our knowledge, is the first to examine trends in digital health technology use among older adults with cancer and identify factors associated with digital health use using nationally representative cohort data. Our study observed a sturdy growth of digital health technology use among older adults with cancer in 4 years before the COVID-19 pandemic (2015-2019) and a strong increase during 2 years of the COVID-19 pandemic (2020-2021); however, the overall prevalence of digital health technology use is relatively low (range 36%-52%) considering its substantial benefits for improved health outcomes. In addition, this study demonstrated that lower digital health use was significantly associated with socioeconomic disparities, fewer comorbidities, and lower physical function.

Recent studies have shown an increased use of digital health technology in both cancer survivors [18,29] and older adults [30]. This study identified a notable increase in the use of digital health technology in older adults with cancer from 2015 to 2021, which is aligned with the report of increased mobile technology device ownerships in older adults from 30% in 2015 to 61% in 2021 [30]. Furthermore, there was a strong increase in digital health technology use after the COVID-19 pandemic (ie, 51% in 2020 and 52% in 2021) compared with that before the pandemic (ie, 45% in 2019). The rise in digital health technology use before and after the COVID-19 pandemic corresponds with previous studies focusing on the general population of older adults [31,32].

When seeing each type of digital health technology use, obtaining health information, contacting clinicians, and filling prescriptions were found out to be the most prevalent reasons for technology use. These findings may be related to a rapid scaling up of telemedicine adoption due to the impact of the pandemic that restricted in-person communication with health care team members [33-36]. On the other hand, handling insurance was the least prevalent reason for technology use in the entire follow-up period. Prior research suggests that caregivers typically manage insurance-related issues for older adults [37-39]. Therefore, this result should be interpreted with caution, as it might not directly reflect the respondents’ personal experiences in handling insurance matters using technology.

In this study, the prevalence of digital health technology use in older adults with cancer was lower than the prevalence of everyday technology use (ie, 36%-52% vs 59%-69%). It may be understood that older adults with cancer have sufficient materials or capabilities to use everyday technology, but they are less likely to use digital health technology. One of the multiple potential factors associated with the lower use of digital health technology in older adults with cancer may be that their need for digital health technology is lower than everyday technology, or their low eHealth literacy [40], which refers to an individual’s ability to use health-related information on electronic devices [41]. Hoogland et al [40] found that older adults with cancer were less likely to be capable in seeking health-related information on the internet, although most of them who participated in the study had an active email address or wearable devices to track activities daily. Nonetheless, caution in interpreting this relationship is advisable, as reduced usage may not necessarily indicate diminished needs. Such findings may suggest the necessity of exploring perceived barriers about digital health technology use by older adults with cancer.

Socioeconomic and racial disparities in cancer survivors’ digital health technology use were seen in older adults with cancer. This study revealed that older adults with cancer who were of White race and reported higher income and education levels were more likely to use digital health technology, which is congruent with a body of literature that examined such disparities in older adults or cancer survivors in all age ranges [13,29,42,43]. In prior studies, older age was one of the most significant factors associated with low use rate of digital health technology along with lower income and education levels and being non-White race [13,29,42,43]. In this study, there was the same association of low income and education levels and being non-White race with low use rate of digital health technology among older adults with cancer. These findings may suggest that the digital divide exists in older adults with cancer. The digital divide refers to the gap between having access to technology and not having access to it [44]. The digital divide can be caused not only by technology ownerships and broadband data access but also by trust between patients and providers and confidence in using health-related technology [44,45]. Although it is unclear that closing the digital divide is directly associated with increased engagement in digital health technology use, there is a lack of knowledge regarding how to address digital divide in the innovation and development of digital health technology. The needs of older adults with cancer for digital health technologies may be better understood through further study in order to identify methods to lessen such technological disparities.

In this study, older adults with cancer with more comorbidities were more likely to use digital health technology after controlling for all other variables. Congruent with our finding, previously published literature showed more electronic communication use in the general population, including patients without cancer, with more comorbidities [13]. However, in Cho et al’s study [18], patients with and survivors of cancer’s use of electronic communication with health care providers was not significantly associated with the number of comorbidities. The inconsistent findings may be explained by the difference in age groups. Older adults with cancer experience more comorbidities, which may generate higher needs for health-related information seeking [46]. However, such inconsistency may also be interpreted by the evidence that showed cancer survivors’ decreased demands for seeking health-related information in online when they had severe comorbidities [46,47]. Further research about older cancer survivors’ various needs for health-related information that may differ by their number and types of comorbidities and their needs for technology should be conducted.

Older adults with cancer in this study who had a cognitive problem (ie, the diagnosis of dementia) and lower physical function (ie, gait speed) were less likely to use digital health technology. This finding may indicate that frailty is particularly associated with older cancer survivors’ low prevalence of digital health technology use. The concept of “frailty,” which refers to a decline in multiple physiologic systems that can result in disabilities and vulnerabilities, is emerging in oncology research and practice as the proportion of older cancer survivors increases [48,49]. Our finding may indicate the need for a unique approach to older cancer survivors who are frail or prefrail in order to adapt shifting trends in digital health technology use for cancer survivorship care.

This study has several limitations. First, from 2015 to 2021, many survey participants were lost to follow-up or died. Of the 1996 participants, 621 participants were deceased between round 5 and round 11 and 413 participants were lost to follow-up. The sample may not be representative of the general cancer survivor population due to dropout and nonresponse. However, we controlled for the nonresponse rate and weights in our analysis to reduce the bias; second, the frequency of technology use and cancer-specific information were not available in the data set. Participants who use digital health technology may have a difference in frequency and pattern of use. Third, because of the cross-sectional nature of the analysis to examine the association between potential factors and digital health technology use, we could not clarify the direction of these relationships. For example, using digital health may help improve the physical function of older cancer survivors.

### Conclusions

This study described digital health or everyday technology use in older adults with cancer from 2015 to 2021 and the associated factors with the prevalence of digital health technology use in the prepandemic period. Our findings indicated that there had been a gradual increase in technology use in older adults with cancer, particularly during the COVID-19 pandemic. However, the overall prevalence of digital health technology usage remains relatively low despite its significant potential for enhancing health outcomes. Furthermore, our study highlighted that reduced digital health adoption was associated with socioeconomic inequalities, a lower number of comorbidities, and diminished physical function. As the proportion of the older population rises in cancer survivorship, such findings may imply that future developments in digital health technology should take into account the needs of older adults with cancer, including declined health status and frail health conditions, for widespread and consistent use. Future studies are required to examine the unique clinical and physical traits of older cancer survivors and approaches to integrating digital health technology into their cancer survivorship care delivery.

## Appendix

Multimedia Appendix 1 Weighted estimates of odds ratio in logistic regression models, 2019 and 2021.

## Abbreviations

ADL: activities of daily living
NHATS: National Health and Aging Trends Study
SPPB: Short Physical Performance Battery
OR: odds ratio
