# Supplementary material for: Use of Digital Health Technology Among Older Adults With Cancer in the United States: Findings From a National Longitudinal Cohort Study (2015-2021)
Source: J Med Internet Res. 2023 May 31;25:e46721. doi: 10.2196/46721 (PMC10267783; doi:10.2196/46721)
Supplement: Multimedia Appendix 1 [file jmir_v25i1e46721_app1.docx]

**Multimedia Appendix 1. Weighted estimates of odds ratio in logistic regression models, 2019 and 2021**

|  | Any Use of Digital Health  (year 2019) | Any Use of Digital Health  (year 2021) |
| --- | --- | --- |
|  | Adjusted OR (95% CI) | Adjusted OR (95% CI) |
| Age (y) |  |  |
| 65-69 | Reference | Reference |
| 70-74 | **0.36 (0.10, 1.23)** | -- |
| 75-79 | **0.25 (0.09, 0.69)** | **0.50 (0.27, 0.93)** |
| 80-84 | **0.19 (0.07, 0.57)** | **0.32 (0.15, 0.69)** |
| 85-89 | **0.14 (0.05, 0.40)** | **0.22 (0.10, 0.49)** |
| 90- | **0.10 (0.03, 0.34)** | **0.25 (0.10, 0.59)** |
| Female | **1.72 (1.15, 2.57)** | **1.79 (1.01, 3.16)** |
| Race/ethnicity |  |  |
| White | Reference | Reference |
| Black | **0.56 (0.32, 0.99)** | 0.68 (0.42, 1.09) |
| Hispanic | **0.22 (0.06, 0.84)** | **0.17 (0.03, 0.86)** |
| Other | 0.65 (0.20, 2.13) | 0.65 (0.18, 2.32) |
| Married or partnered | 1.11 (0.74, 1.67) | 1.33 (0.78, 2.25) |
| Education level |  |  |
| Less than high school | **0.14 (0.08, 0.27)** | **0.11 (0.05, 0.25)** |
| High school graduate | **0.26 (0.17, 0.40)** | **0.28 (0.17, 0.47)** |
| Some college | **0.39 (0.26, 0.60)** | **0.56 (0.35, 0.89)** |
| College graduate or higher | Reference | Reference |
| Annual income ($) |  |  |
| <15000 | Reference | Reference |
| 15000-29999 | 1.29 (0.50, 3.30) | 1.94 (0.53, 7.02) |
| 30000-44999 | 2.06 (0.86, 4.92) | 2.77 (0.69, 11.08) |
| 45000-60000 | **2.32 (0.84, 6.44)** | **4.71 (1.44, 15.39)** |
| >60000 | **3.41 (1.32, 8.85)** | **7.18 (1.82, 28.31)** |
| Self-rated health |  |  |
| Excellent | Reference | Reference |
| Very good | 0.93 (0.50, 1.74) | 0.66 (0.34, 1.27) |
| Good | 1.18 (0.67, 2.09) | 0.61 (0.30, 1.25) |
| Fair | 0.96 (0.46, 1.98) | 0.75 (0.34, 1.66) |
| Poor | 1.18 (0.36, 3.83) | 0.53 (0.17, 1.63) |
| No. of comorbidities | **1.16 (1.01, 1.33)** | 1.09 (0.96, 1.24) |
| No. of ADL limitations | 1.14 (0.99, 1.30) | 1.17 (1.00, 1.37) |
| Dementia | **0.35 (0.14, 0.90)** | **0.51 (0.28, 0.92)** |
| Anxiety and depression | 0.95 (0.87, 1.03) | 0.99 (0.90, 1.08) |
| SPPB score | **1.13 (1.06, 1.22)** | **1.12 (1.02, 1.24)** |
| Grip strength score | 1.01 (0.86, 1.18) | 1.05 (0.85, 1.30) |

*Note*. Models were adjusted for complex survey design. Bolded values mean p<.05. Abbreviations: CI, confidence intervals; OR, odds ratio; ADL, activities of daily living, SPPB, short physical performance battery.
